# Physical Activity and Cardiac Morphologic Adaptations

**DOI:** 10.31083/j.rcm2405142

**Published:** 2023-05-11

**Authors:** Andreas Pittaras, Charles Faselis, Michael Doumas, Charalampos Grassos, Peter Kokkinos

**Affiliations:** ^1^Department of Cardiology, Washington DC Veterans Affairs Medical Center, Washington, DC 20422, USA; ^2^School of Medicine and Health Sciences, George Washington University, Washington, DC 20052, USA; ^3^Department of Kinesiology and Health, School of Arts and Sciences, Rutgers University, New Brunswick, NJ 08901, USA

**Keywords:** exercise, physical activity, left ventricular mass, cardiac structure, cardiac function, arrhythmias, athletes

## Abstract

Chronic and intense exercise programs lead to cardiac adaptations, followed by increased left ventricular wall thickness and cavity diameter, at times meeting the criteria for left ventricular hypertrophy (LVH), commonly referred to as “*athlete’s heart*”. Recent studies have also reported that extremely vigorous exercise practices have been associated with heightened left ventricular trabeculation extent, fulfilling noncompaction cardiomyopathy criteria, as part of exercise-induced structural adaptation. These changes are specific to the exercise type, intensity, duration, and volume and workload demands imposed on the myocardium. They are considered physiologic adaptations not associated with a negative prognosis. Conversely, hypertrophic cardiac adaptations resulting from chronic elevations in blood pressure (BP) or chronic volume overload due to valvular regurgitation, lead to compromised cardiac function, increased cardiovascular events, and even death. In younger athletes, hypertrophic cardiomyopathy (HCM) is the usual cause of non-traumatic, exercise-triggered sudden cardiac death. Thus, an extended cardiac examination should be performed, to differentiate between HCM and non-pathological exercise-related LVH or athlete’s heart. The exercise-related cardiac structural and functional adaptations are normal physiologic responses designed to accommodate the increased workload imposed by exercise. Thus, we propose that such adaptations are defined as “eutrophic” hypertrophy and that LVH is reserved for pathologic cardiac adaptations. Systolic BP during daily activities may be the strongest predictor of cardiac adaptations. The metabolic demand of most daily activities is approximately 3–5 metabolic equivalents (METs) (1 MET = 3.5 mL of $O_{2}$ kg of body weight per minute). This is similar to the metabolic demand of treadmill exercise at the first stage of the Bruce protocol. Some evidence supports that an exercise systolic BP response ≥150 mmHg at the end of that stage is a strong predictor of left ventricular hypertrophy, as this BP reflects the hemodynamic burden of most daily physical tasks. Aerobic training of moderate intensity lowers resting and exercise systolic BP at absolute workloads, leading to a lower hemodynamic burden during daily activities, and ultimately reducing the stimulus for LVH. This mechanism explains the significant LVH regression addressed by aerobic exercise intervention clinical studies.

## 1. Introduction

**Fig. 1.**
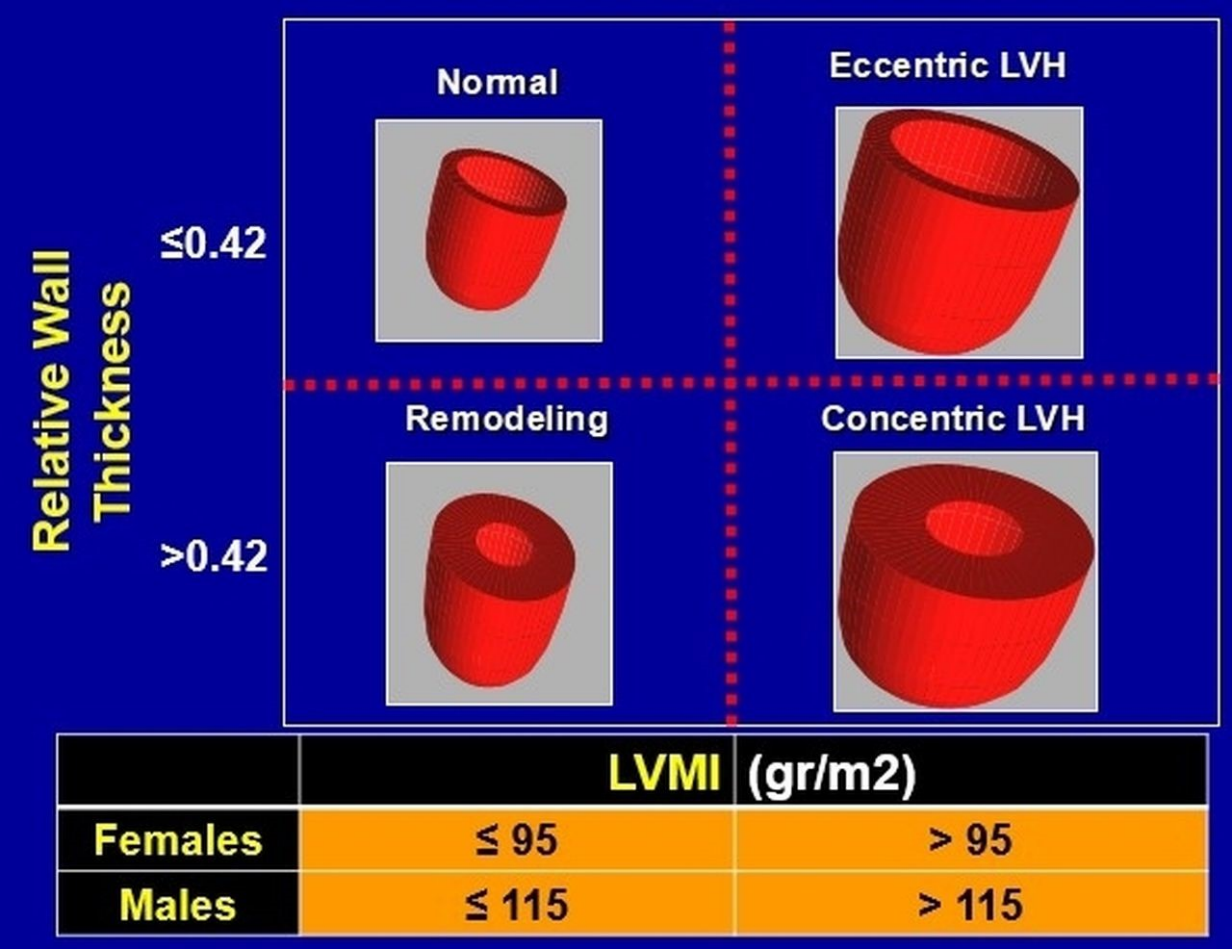
**Classification of all left ventricular hypertrophy patterns, based on calculated relative wall thickness and left ventricular mass index (LVMI)**. LVH, left ventricular hypertrophy; LVMI, left ventricular mass index.

Chronic exaggerated increases in the hemodynamic load, lead to balancing responses and changes in cardiac myocytes, typically leading to an increase in left ventricular mass (LVM) and finally in established left ventricular hypertrophy (LVH) [1]. Specifically, chronically elevated blood pressure (BP) is likely to result in increased ventricular wall thickness and left ventricular mass index [(LVMI), (>95 gr/$m^{2}$ for females and >115 gr/$m^{2}$ for males)], and reduced left ventricular cavity size, a pattern known as concentric LVH [2, 3, 4, 5]. A more specific definition of concentric LVH is also defined as the ratio of the left ventricular wall thickness to end-diastolic diameter >0.42, known as “relative wall thickness” [6]. Contrary, volume overload is accompanied by eccentric LVH characterized by increased LVMI, relatively large left ventricular cavity size and normal relative wall thickness (≤0.42) [7, 8]. A third pattern has also been described, defined by an increase in relative wall thickness, but not LVMI, known as concentric remodeling [8, 9, 10]. All cardiac hypertrophy patterns and cardiac parameters (relative wall thickness and LVMI for both genders) are presented in Fig. 1.

The presence of LVH, especially the concentric geometry pattern is a strong and independent predictor of cardiovascular events and all-cause mortality. The risk of cardiac events, as well as sudden cardiac death, increases three-fold in this population [4, 11, 12]. Conversely, LVH regression, the outcome of resting BP reduction and hypertension control achieved by most antihypertensive medications, is associated with a significant reduction in cardiovascular events and death [13, 14, 15, 16]. The degree of LVH regression is strongly related to the degree of BP reduction, supporting partly the pathophysiologic mechanism of the stimulus role of pressure overload in the development of concentric LVH [17, 18, 19, 20].

Although the exercise-related favorable health outcomes have been described extensively [21] the exercise-related BP management in hypertensive patients with Stage II hypertension is less known. In addition, the impact of exercise training on BP response during physical work or exercise and its association with cardiac structure and function is also poorly understood [22]. In our previous work we have provided evidence indicating that exercise BP response during relatively low workload of 3–5 metabolic equivalents (METs; 1 MET = 3.5 mLof oxygen consumed per kg of body weight per minute) may be a strong indicator of LVH. Furthermore, achieving lower exercise BP at an absolute and relative workload may lead to LVH regression [23, 24]. In this review, we summarize the findings of select studies on the association between exercise and cardiac structural changes and their clinical significance. We also present evidence on the exercise-BP-LVH interaction, and LVH regression associated with proper exercise training.

## 2. The Athlete’s Heart: Historical Point of View 

Temporary sudden or long-standing increases in physical workload or exercise also pose an increased hemodynamic demand on the cardiovascular system. Subsequently, appropriate, acute, and chronic cardiac adaptations occur to accommodate this increased workload.

In 1975, Morganroth and colleagues [25] described 2 distinctly different cardiac morphological adaptations in athletes, as the outcome of the specific hemodynamic load imposed on the ventricles during repeated exercise bouts of different exercise modes. In general, they reported that left ventricular end-diastolic and cardiac mass were increased in athletes engaging in repetitive isotonic contractions such as running, and normal wall thickness (≤12 mm). Athletes engaging in isometric or resistance type activities such as wrestling and shot putting, exhibited normal left ventricular end-diastolic volume, but increased wall thickness (13–14 mm). These morphological cardiac changes reflect the specific demand imposed by the cardiovascular system by the type of exercise. During repetitive and prolonged muscular contractions of relatively low-to-moderate intensity (aerobic exercise training), end-diastolic volume is increased with each cardiac cycle, a consequence of increased venous return while afterload decreases due to the exercise-related vasodilatory response. Prolonged and repetitive exposure to this volume overload leads mainly to an increase in the left ventricular (LV) cavity dimension referred to as “eccentric” cardiac hypertrophy. In contrast, strenuous resistive exercise (strength training) poses a pressure overload, the consequence of increased afterload, possibly due to the arterial compression with each muscular contraction. Prolonged exposure to this increased pressure leads to a concentric form of hypertrophy, characterized by increased left ventricular wall thickness with no significant change in cavity size.

Although this hypothesis has been challenged recently, the general concept that cardiac adaptations reflect the demand imposed the exercise type remains. However, it should be emphasized that most physical activities combine a static and an isotonic component. Thus, cardiac adaptations are more diverse and can consist of concentric and eccentric morphology which can coexist to accommodate the imposed exercise demands on the cardiovascular system [26, 27].

Exercise-related cardiac alterations, including hypertrophy, dilatation, bradycardia, and arrhythmias coming from chronic physical activity experienced by athletes have been reported more than two centuries ago, and continue to be of interest to physicians and scientists. Early cases of an enlarged heart (end of 19th century) were reported in Harvard University rowers [28], elite Nordic skiers [29], and Boston Marathon runners [30, 31], mostly viewed as useful modifications in response to exercise [30, 31]. The evolution of the electrocardiogram (ECG) revealed cardiac hypertrophy abnormalities in the electrical activity of the heart [32, 33, 34, 35, 36], while advances in echocardiography and magnetic resonance imaging have led to a better understanding of the athlete’s heart. An important study on the long-term consequences of endurance training in Olympic athletes reported no adverse cardiac events during 8.6 ± 3 years of intense training [37]. Generally, the predominant conception is that the cardiac anatomical and functional changes resulting from long-term, intense, but not unjustified exercises are considered normal adaptations, without unfavorable cardiac events of impaired cardiac function [37].

As mentioned, exercise-induced anatomical and functional cardiac changes are specific to the type and intensity of the activity. Accordingly, acute hemodynamic cardiovascular responses to these two types of exercise differ markedly. Therefore, long-term exposure to either of them is likely to lead to specific chronic cardiovascular adaptations to accommodate the specific demands imposed on the cardiovascular system by the exercise type or physical work.

## 3. Aerobic Exercises and LVH

Some studies reported that the upper limits of wall thickness resulting from engaging in aerobic exercise regularly to be ≤13.0 mm. However, wall thickness ≥13 mm has been reported in a small number of highly-trained athletes. In a study of 947 Italian elite Olympic athletes exposed to intense exercise training of both isotonic and isometric/resistance exercises, left ventricular wall thickness exceeded 13 mm in only 15 rowers and 1 cyclist (1.7%) [38]. Additionally, in 3000 highly trained British athletes, only 1.5% presented increased left ventricular wall thickness of more than 13 mm, with mild chamber enlargement [39]. Finally, wall thickness exceeding 13 mm has been reported in a small number of athletes who engage in extreme exercise programs such as ultra-distance marathon races and highly trained cyclists [40, 41, 42, 43]. Collectively, these findings suggest that “physiological” limits of exercise-related left ventricular wall thickness may exceed 13 mm and go as high as 16 mm in a small number of athletes engaging in activities that require extreme efforts and beyond the capacity of most individuals (Table 1) [26, 27, 38, 44, 45, 46]. However, the relatively small number of individuals with such a cardiac morphology caution against definitive conclusions. Finally, cardiac wall thickness >16 mm is considered pathological (Table 1) [27, 46, 47, 48, 49, 50].

**Table 1.** **Risk stratification based on Left ventricular wall thickness**.

Left ventricular wall thickness		
≤13 mm for males	>13–16 mm for males	>16 mm
≤12 mm for males	>12 mm for females	
Gender	Family history	Family history
Race	Gender	Gender
Exercise type	Exercise type	Exercise type
Cardiac function	Symptoms	Symptoms
	Cardiac function	Cardiac function
	Asymmetrical LVH	Asymmetrical LVH
	Specific echocardiographic findings	Specific echocardiographic findings
	Specific ECG findings	Specific ECG findings
	Cardiac Echocardiographic findings	Cardiac Magnetic Resonance Imaging
	Detraining LVH regression	Detraining LVH regression
No further evaluation in most cases	Cardiac evaluation in many	Cardiac evaluation in all

Left ventricular wall thickness thresholds and need for further cardiac evaluation, based on demographic, clinical and cardiac imaging criteria. LVH, left ventricular hypertrophy; ECG, electrocardiographic.

Female athletes usually have smaller frame, lower lean body mass, different hormonal profile, and lower peak exercise systolic BP, stroke volume and ${\mathrm{VO}}_{2}$, affecting their cardiac dimensions [51, 52, 53]. All the above may impact differently on cardiac structural adaptations resulting from intense training. In a population of 600 elite female athletes, none had LV wall thickness >12 mm [54]. Similarly, LV wall thickness was ≤12 mm in all 438 white female elite athletes [55]. In this isotonic exercise subgroup, eccentric LVH was mainly observed in females with none having relative wall thickness (RWT) >0.48 or LVM >145 g/$m^{2}$. In comparison, males exhibited more frequently concentric remodeling LVH. Finally, Rawlins *et al*. [56] studied 200 black female athletes and compared them to matched white female athletes. Cardiac wall thickness >11 mm was found in 3% of black athletes, compared with none of white female athletes. None exhibited LV wall thickness >13 mm. The findings of these studies suggest that cardiac structural changes in female athletes are mostly characterized by an increase in chamber size. Thus, the presence of concentric hypertrophy should be evaluated carefully (Table 1) to rule-out the presence of hypertrophic cardiomyopathy.

In conclusion, vigorous exercise-related chronic cardiac adaptations are considered “physiologic” responses to the specific hemodynamic load imposed to of the particular sport, exercise, or physical activity. These adaptations are not associated with diastolic dysfunction, atrial or ventricular complex arrhythmias, or worse prognosis, conditions observed in hypertension-related LVH [7, 8]. Additionally, there is evidence that exercise-induced LVH regression is observed after 3 or more months of exercise training discontinuation [46, 50], further supporting the concept that the cardiac adaptations are in response to an increased workload and not pathological.

## 4. Resistance Exercises and LVH 

**Fig. 2.**
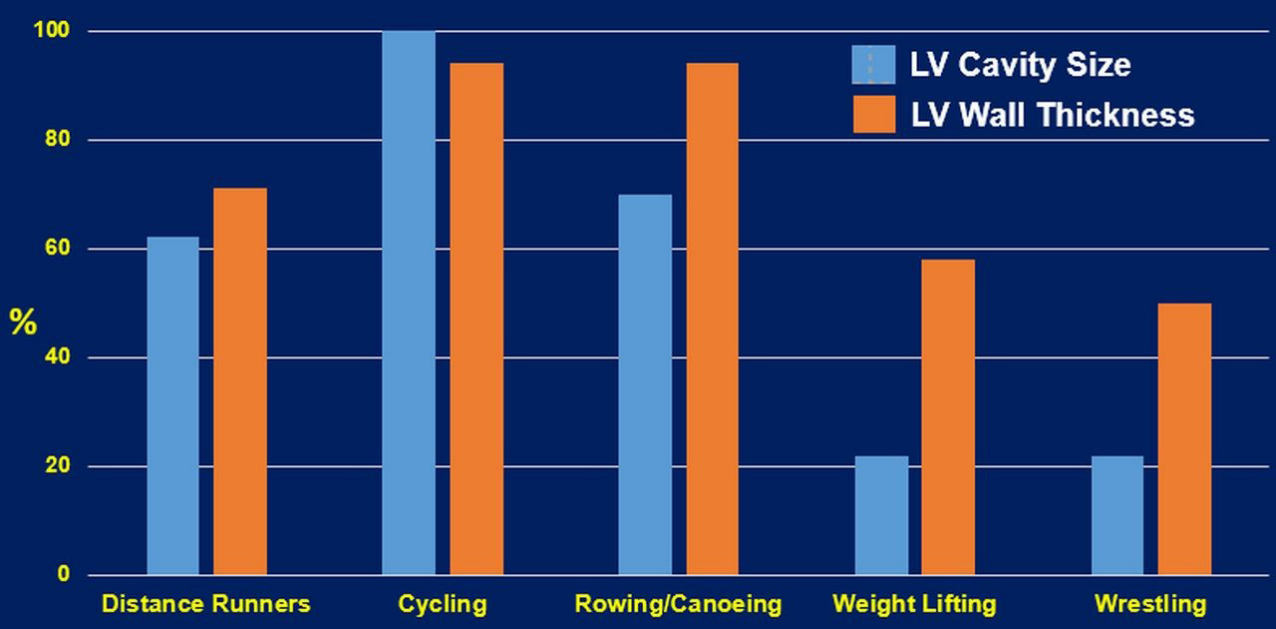
**Cardiac structural changes of select sports that represent aerobic, resistance and the combination of the two exercise types [27]**. LV, left ventricular.

Resistance exercises are typically accompanied by increased ventricular wall thicknesses, asymmetrically to chamber inner dimensions. Whether the concentric LVH is induced by resistance training alone is debatable [32]. Most sports or daily activities contain both aerobic and anaerobic types of exercises. Therefore, cardiovascular adaptations are likely to reflect the combined types of the workload of the sport or activity, leading to mixed cardiac remodeling patterns. This is supported by the finding of elite heavy-trained athletes engaging in sports such as cycling, rowing, and swimming, are a typical example of combined both aerobic and resistance exercises. These athletes (Fig. 2) often present the most excessive increase in all left ventricular geometry parameters (wall thickness and cavity dimension) [36]. Finally, it is important to emphasize that an increase in wall thickness or LV diastolic dimension alone should not be considered a favorable physiological adaptation. LV dilatation without concomitant wall hypertrophy will lead to an undesirable increase in wall tension that is detrimental to the heart [50]. Such conditions are usually observed in patients with chronic heart failure.

## 5. Exercise-Induced LVH and Provocation of Arrhythmias

There is no consensus as to whether adaptations observed in the cardiac architecture and function in athletes are considered favorable or at least benign to the cardiovascular system or pathological changes favoring increased risk of arrhythmias. Provocation of exercise-related complex arrhythmias in certain situations [50] and a higher incidence of atrial fibrillation (AF) in middle-aged and older heavy-trained athletes engaged in chronic high-intensity exercises, as compared to non-athletes have been reported [57, 58, 59, 60, 61, 62, 63]. This relationship does not appear to be directly related to the amount of exercise-related physiologic LVH [49, 64]. It is more directly related to intensity as well as the duration of strenuous exercises [57, 58, 61, 63].

**Fig. 3.**
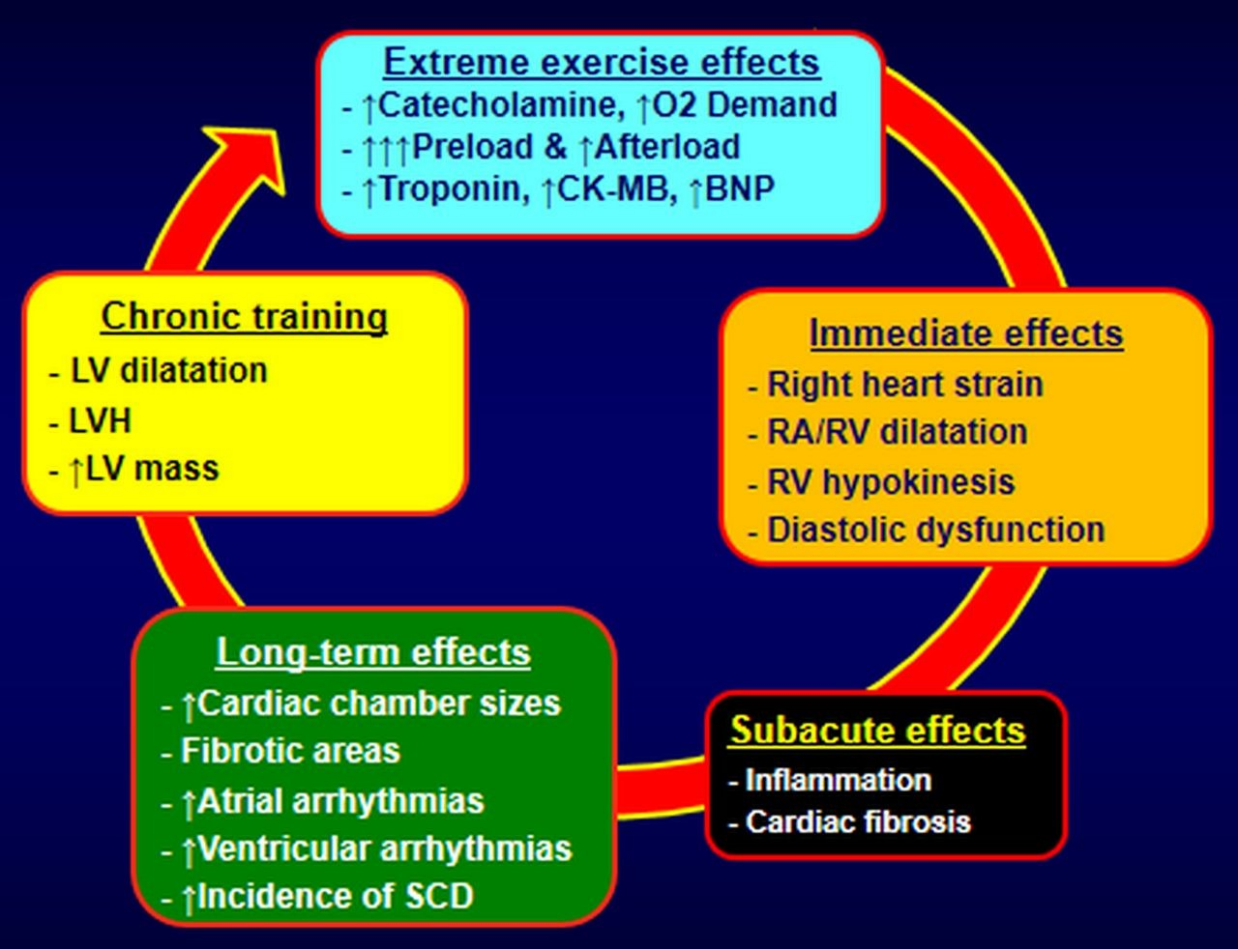
**Proposed pathophysiologic mechanisms of cardiomyopathy and arrhythmias in endurance athletes**. LVH, left ventricular hypertrophy; LV, left ventricular; CK-MB, creatine kinase-myocardial band; BNP, B-Type natriuretic peptide; RA/RV, right atrium/right ventricle; SCD, sudden cardiac death.

Whether exercise-related atrial arrhythmogenicity and chaotic ectopy from the pulmonary veins, are the main cause of atrial arrhythmias, or whether other pathophysiologic mechanisms are responsible has not been determined. A survey of elite cyclists did not document increased atrial ectopy [65]. Additionally, increased vagal tone is observed in many endurance athletes [60], leading to bradycardia and reduced atrial refractory period, major modulators of heart rhythm, triggering re-entry arrhythmias. It is well-accepted that the pressure in the pulmonary arteries increases during exercise [66]. During intense exercise, higher pressures typically notable in athletes, are measured in the right cardiac chambers, with a continuing decline in right ventricular ejection fraction as the duration of vigorous exercise increases [67]. In case of long-term intense exercise stress and without reasonable recovery time, dilatation of the less-muscular chambers (atria and right ventricle) is observed, leading to some degree of inflammation, minor injury, and fibrotic lesions. These events are the usual pathophysiologic suspects for electrical instability and cardiac complex arrhythmias [68]. The presence of an exercise-related arrhythmogenic right ventricular cardiomyopathy has been the subject of considerable debate in some studies [66, 69]. Finally, another study in elite athletes [70] observed no relationship between ventricular ectopy and the magnitude of exercise induced LVH, indicating the benign nature of ventricular ectopy and the expression of athlete’s heart. Although the proposed pathogenic pathways for the development of exercise-related cardiomyopathy, summarized in Fig. 3 are noteworthy, no established pathophysiologic mechanism exists to explain any relationship between high exercise intensity and risk of AF.

Nevertheless, such arrhythmias rarely have been associated with adverse cardiac events and are usually disappeared or to a large degree reduced after reasonably short periods of deconditioning [49].

We examined the role of cardiorespiratory fitness (CRF) on new-onset AF in 5962 middle-aged and older Veterans. Our findings showed an inverse and graded relationship between CRF and AF risk. The AF incidence was 21% lower for each 1-MET increase in exercise capacity. Compared with the least fit individuals, the AF-risk was 20%, 45%, and 63% lower for moderately fit, fit, and highly fit individuals, respectively [71]. Recently we reported comparable findings (ACC 2022-Highlights) in a large cohort of 459,592 hypertensive veterans, showing an independent, inverse, and graded association between CRF and AF-risk [72]. Compared with the least fit (4.5 METs) hypertensives (reference group), the AF-risk was 39% lower for moderately fit (8.3 METs), 48% for fit (10.3 METs), and 55% for highly fit (13.1 METs). Similar findings were observed in both, younger and older than 65 years, suggesting that CRF achieved by moderate or even high-intensity exercise programs, protects from the risk of new onset AF, regardless of age.

## 6. Exercise Training, BP Response and LVH Regression

Relatively small reductions in BP achieved by antihypertensive therapy (even 5 mmHg), lead to impressive beneficial effect, including mortality and morbidity risk reduction [73, 74]. LVH regression comparable to the degree of BP reduction [17, 18, 19, 20] is responsible in part for the health benefits [13, 14, 15, 16]. Several well-controlled studies [22, 75, 76, 77, 78] have shown significant exercise-related BP reduction in both systolic and diastolic BP (4–10 mmHg and 3–8 mmHg respectively), independent of age, gender, and weight loss. Therefore, it is reasonable to assume that similar exercise-related reductions in BP should yield similar health outcomes, including the effects of exercise-induced LVH regression. This assumption is supported by the findings of large and well-designed epidemiologic studies that have shown a significant, inverse, and graded relationship between CRF (expressed by exercise capacity), and mortality risk in hypertensive and pre-hypertensive individuals [21, 79, 80, 81, 82, 83].

LVH regression resulting from exercise-related BP reduction has not been evaluated extensively. However, most exercise studies in patients with LVH confirm the beneficial effect of lowering BP by exercise, in this phenotype of target organ damage [84, 85, 75, 76, 77, 78].

We studied 46 men with resistant hypertension under multi-drug therapy (57 ± 10 years of age) engaged in 16 weeks of supervised aerobic exercise program (stationary bicycle), in combination with antihypertensive drugs or antihypertensive drugs alone. After 16 weeks of training, we noted a reduction in systolic and diastolic BP of 7 mmHg and 5 mmHg, respectively, whereas diastolic BP in the reference group increased by 2 mmHg. Additionally, in the exercise group, LV wall thickness and the LVMI decreased significantly, in comparison to the reference drugs alone group [22]. Others performed an exercise training program for 15.7 ± 5.8 months (3 times per week, aerobic, stationary bicycle and muscular relaxation), in 17 patients with mild hypertension, nonresponders to a low sodium diet. Compared with 15 untrained matched patients, there were significant BP decreases, with additional trend to LVMI decrease [86]. In another study of 82 overweight individuals were in (1) supervised aerobic exercise-only group, (2) a behavioral weight loss program, including exercise, and (3) reference group. After 6 months of follow-up, BP decreased by 7/6 mmHg for systolic/diastolic BP, respectively, in the weight management plus exercise group, and by 3/4 mmHg in the aerobic exercise group. Furthermore, the intervention groups, compared with the reference group, had additional benefits in impaired cardiac structure, with a decrease in LV wall thickness and a trend in LVMI [87]. Significant benefits in impaired cardiac geometry were also reported in hypertensive patients after 6 months of aerobic exercise program [88], and a trend for LVM in middle-aged hypertensive individuals of both genders [89]. Comparably, another study reported a notable reduction in resting systolic BP, LV wall thickness, and LVMI (partial regression of LVH and LV concentric remodeling), in 11 older (65.5 ± 1.5 years) hypertensives, participating in approximately 6 months of aerobic exercise program [88]. Lastly, in the HARVEST study [90], including 454 stage I hypertensives (18–45 years old), BP dropped with a lower incidence of developing LVH in exercisers (n = 173), compared to physically inactive individuals (n = 281), after a follow-up of 8.3 years.

Contrary to these findings, in a study including 23 obese individuals with high-normal BP, no LVM regression was reported, regardless of the notable reduction in BP [89]. There were also no beneficial effects on cardiac geometry or LV diastolic function, in 51 overweight and obese individuals (BMI: 29.5 ± 4.4 kg/$m^{2}$) with untreated hypertension (63.6 ± 5.7 years), after 6 months aerobic exercise program, compared to usual care (n = 53) [91]. However, the small number of participants, the different types of exercise, and the inclusion of subjects without established hypertension and LVH in the two aforementioned studies [89, 91] constitute major limitations and render their results questionable.

It is reasonable to assume that exercise training or other lifestyle changes (as complementary “interventional therapy”), will not “repair” what is not damaged. The exercise groups were engaged in a combination of both muscle strength and aerobic training, with different long-term cardiac reactions and structural alterations, leading often to undesirable impaired cardiac geometry and progression to LVH [38]. Overall, despite the small body of knowledge and poor quality of data on exercise-related LV structural adaptations, LVH regression is achieved (mostly in individuals with LVH), by engaging in an appropriate type of aerobic exercise training.

## 7. Exercise Blood Pressure and LVH

The current evidence supports a strong, and direct association between the grade of LVH regression and the level of BP reduction with antihypertensive drugs [17, 18, 19, 20]. A meta-analysis of 4 echocardiographic studies, following 1064 hypertensive individuals for 3–10 years (45–51 years of age, 59% men), noted only 8% LVH regression with parallel cardiovascular risk reduction [19]. Another meta-analysis including 80 trials, with 3767 patients in the treatment group and 346 patients in the placebo group, showed a wide spectrum of LVM reduction among antihypertensive agents (6%–13%). Specifically, the greater LVM reduction was induced by renin-angiotensin-aldosterone-system (RAAS) inhibitors and calcium channel blockers (10%–13%), while a smaller reduction was seen by beta-blockers (6%) and diuretics (8%) [92]. The degree of exercise-related LVH regression is analogous to what has been reported by most antihypertensive drugs [93]. Additionally, the observed exercise-related reduction in BP was significantly lower (average 7/5 mmHg in our studies and similar in others) [22, 75, 76, 77, 78], compared with the overall drug-induced BP reduction (26.6/16.6 mmHg) [89]. Despite the significant BP difference, a 12.3% reduction in LVMI has been reported by exercise studies, which is comparable to the reduction obtained by RAAS blockers (13% with angiotensin receptor blockers (ARBs) and 10% with angiotensin-converting enzyme (ACE)-inhibitors), and clearly greater than that realized by beta-blockers (6%) [92]. The BP reduction in the drug-related LVH regression studies, was roughly 13%, representing 1% LVMI regression for every 1% reduction in BP. In our study [22], supervised aerobic exercise training by stationary bicycle, reduced systolic BP by 5% and LVMI by 12.3%, a 2.5% LVMI reduction per 1% reduction in BP. In subsequent studies, our findings suggest that the degree of LVH regression may not be the sole outcome of the exercise-related reduction in resting BP but also the lower exercise systolic BP response at absolute workloads. Specifically, we noted that the systolic exercise BP following 16 weeks of aerobic training was 14% systolic BP reduction at 3 METs, 15% at 5 METs and 9% at peak exercise [22, 94, 95]. Interestingly, the metabolic demand of most daily activities, is equivalent to 3–5 METs [27, 28]. These findings suggest that the hemodynamic load was reduced during daily activities. Additionally, the daily hemodynamic load may be an important predictor of LVH regression, stronger than the resting BP. This hypothesis is supported further by our findings, in 790 prehypertensive men and women (BP <140/90 mmHg). In this study, the strongest predictor of LVH was the exercise systolic BP at approximately 4–5 METs (Stage I of the Bruce protocol), while the predictive value of resting systolic BP was substantially lower [23]. Additionally, we observed a significant inverse relationship between fitness and LVMI, significantly lower exercise systolic BP, and lower LVMI in in subjects with moderate and high exercise capacity. Ambulatory daytime BP, and submaximal exercise BP response at 4–5 METs was also significantly lower in these subjects [24]. Furthermore, for comparable resting BP levels, we defined a systolic BP threshold of ≥150 mmHg at submaximal exercise (4–5 METs), and an ambulatory daytime systolic BP threshold of >140 mmHg beyond which the risk for LVH and impaired cardiac structural parameters, dramatically increased (2-fold and 2.2-fold for every 5 mmHg increase in systolic BP respectively). These BP thresholds were strong predictors of LVH presence with a high sensitivity (88% and 85% respectively) and specificity (74% and 73% respectively), substantially higher than the 6–53% sensitivity of any electrocardiographic parameter alone or in combination [4] (Table 2, Ref. [4, 23]). Additionally, the risk for LVH was reduced by 42% for every 1 MET increase in exercise capacity. Others, in a smaller study in hypertensives (n = 49), observed comparable results, indicating a strong direct association with exercise SBP response at a workload of 7 METs, and all structural parameters of LVH. More specifically, the SBP response at 7 METs was a stronger predictor than the office BP and 24 h ambulatory SBP monitoring [24].

**Table 2.** **Predictive value of exercise at 5 METs and ambulatory daytime SBP, for LVH development [4, 23]**.

	Sensitivity	Specificity
Exercise SBP ≥150 mmHg	88%	74%
Daytime ABP ≥140 mmHg	85%	73%
ECG	6%–53%	89%–100%

Exercise SBP and Daytime ABP at 5 METs provide a better sensitivity, and comparable specificity for prediction of LVH development. SBP, systolic blood pressure; ABP, ambulatory blood pressure; METs, metabolic equivalents; LVH, left ventricular hypertrophy; ECG, electrocardiogram.

Collectively, these findings indicate that aerobic exercise training lowers the exercise systolic BP response during submaximal and peak workloads. The clinical significance of this is that lower BP during daily activities, leads to lower hemodynamic loads daily, subsequently reducing the impetus for LVH development or LVH progression.

## 8. Vigorous Exercise in Patients with Hypertension-Related LVH 

The chronic adaptations of vigorous exercise in competitive (basketball, soccer, football, etc.) and non-competitive athletics (marathon, cycling, weightlifting, etc.) on cardiac structure and function in hypertensives with LVH, are of limited knowledge. Possibly high-intensity exercises impose an exaggerated demand on the cardiovascular system, prolong further abnormal changes, and therefore, are not recommended. Alternatively, all scientific societies recommend a “prescription” of low-to-moderate intensity aerobic exercise training (brisk military walk) of approximately 30–45 minutes per day, 5 days per week, as part of global management [65, 96, 97, 98]. Such an exercise program is safe and feasible for a wide range of ages and hypertensives with co-morbidities [87] and has been shown to have a protective effect on the major cardiovascular risk factors [75], including LVH regression [22].

## 9. Conclusions

Long-term exposure to exercise programs of proper intensity, duration, and volume increases the hemodynamic and cardiac workload. To accommodate this increased demand on the myocardium, cardiovascular adaptations that include increased LV cavity size and LV wall thickness or both ensue. These adaptations are specific to the cardiac demand imposed by the exercise type, intensity, and volume. LV wall thickness observed with prolonged exercises usually does not exceed 13 mm. However, left ventricular wall thickness as high as 16 mm has been reported in some highly trained athletes engaging in extreme exercise practices such as ultramarathon running or a combination of vigorous aerobic and isometric/resistance exercise programs. These adaptations are relatively benign in the absence of hypertrophic cardiomyopathy (HCM) or other cardiac malformations. In these athletes, the clinical distinction between exercise-related “benign” LVH and the presence of HCM, the main reason for sudden cardiac death in apparently healthy athletes, may not be easily discernable. Thus, we strongly recommend that the individuals are examined by a cardiologist with experience in this sports cardiology.

Evidence supports that exercise systolic BP response ≥150 mmHg at the workload of 4–5 METs may represent a threshold for cardiac structural adaptations. Moderate-intensity aerobic training decreases exercise systolic BP at relative and absolute workloads, leading to lower LV workloads and a decrease in the impetus for LVH progression. Current guidelines for optimal exercise-related health benefits advocate ≥150 minutes per week of moderate, intensity aerobic exercise for most middle-aged and older individuals. The exercise recommendations advocated by the majority of scientific societies includes a brisk walk to a slow jog at the exercise intensity of 12–16 minutes per mile, 4–6 days weekly for 150–200 minutes per week. It is strongly recommended that such guidelines are followed and long-term, high-intensity, and high-volume exercises without rest periods between exercising days should be avoided, especially by older, or high-risk populations.

Exercise-related cardiac structural changes that lead to improved cardiac function are physiological and necessary to meet the increased demand posed by exercise. Thus, the exercise-related LVH may be considered as “eutrophic” LVH and reserve “hypertrophic” cardiac adaptations solely those imposed by pathophysiologic mechanisms (hypertension, cardiac injury-fibrosis, HCM) and intrude upon cardiac function, usually leading to “malignant” LVH, cardiac dysfunction, complex arrhythmias and even death.

## References

[1] Lorell BH, Carabello BA (2000). Left ventricular hypertrophy: pathogenesis, detection, and prognosis. *Circulation*.

[2] Cohn JN, Ferrari R, Sharpe N (2000). Cardiac remodeling—concepts and clinical implications: a consensus paper from an international forum on cardiac remodeling. *Journal of the American College of Cardiology*.

[3] Ganau A, Devereux RB, Roman MJ, de Simone G, Pickering TG, Saba PS (1992). Patterns of left ventricular hypertrophy and geometric remodeling in essential hypertension. *Journal of the American College of Cardiology*.

[4] Levy D, Labib SB, Anderson KM, Christiansen JC, Kannel WB, Castelli WP (1990). Determinants of sensitivity and specificity of electrocardiographic criteria for left ventricular hypertrophy. *Circulation*.

[5] Manyari DE (1990). Prognostic implications of echocardiographically determined left ventricular mass in the Framingham Heart Study. *The New England Journal of Medicine*.

[6] Lang RM, Bierig M, Devereux RB, Flachskampf FA, Foster E, Pellikka PA (2005). Recommendations for chamber quantification: a report from the American Society of Echocardiography’s Guidelines and Standards Committee and the Chamber Quantification Writing Group, developed in conjunction with the European Association of Echocardiography, a branch of the European Society of Cardiology. *Journal of the American Society of Echocardiography*.

[7] Frohlich ED, Apstein C, Chobanian AV, Devereux RB, Dustan HP, Dzau V (1992). The heart in hypertension. *The New England Journal of Medicine*.

[8] Drazner MH (2011). The Progression of Hypertensive Heart Disease. *Circulation*.

[9] Khouri MG, Peshock RM, Ayers CR, de Lemos JA, Drazner MH (2010). A 4-Tiered Classification of Left Ventricular Hypertrophy Based on Left Ventricular Geometry. *Circulation: Cardiovascular Imaging*.

[10] Sehgal S, Drazner MH (2007). Left ventricular geometry: does shape matter. *American Heart Journal*.

[11] Verdecchia P, Schillaci G, Borgioni C, Ciucci A, Gattobigio R, Zampi I (1998). Prognostic Value of a New Electrocardiographic Method for Diagnosis of Left Ventricular Hypertrophy in Essential Hypertension. *Journal of the American College of Cardiology*.

[12] Verdecchia P, Schillaci G, Borgioni C, Ciucci A, Gattobigio R, Zampi I (1996). Prognostic value of left ventricular mass and geometry in systemic hypertension with left ventricular hypertrophy. *The American Journal of Cardiology*.

[13] Devereux RB, Wachtell K, Gerdts E, Boman K, Nieminen MS, Papademetriou V (2004). Prognostic significance of left ventricular mass change during treatment of hypertension. *The Journal of the American Medical Association*.

[14] Pierdomenico SD, Cuccurullo F (2010). Risk Reduction after Regression of Echocardiographic Left Ventricular Hypertrophy in Hypertension: A Meta-Analysis. *American Journal of Hypertension*.

[15] Larstorp AC, Okin PM, Devereux RB, Olsen MH, Ibsen H, Dahlof B (2012). Regression of ECG-LVH is associated with lower risk of new-onset heart failure and mortality in patients with isolated systolic hypertension; The LIFE study. *American Journal of Hypertension*.

[16] Okin PM (2004). Regression of Electrocardiographic Left Ventricular Hypertrophy during Antihypertensive Treatment and the Prediction of Major Cardiovascular Events. *The Journal of the American Medical Association*.

[17] Mathew J, Sleight P, Lonn E, Johnstone D, Pogue J, Yi Q (2001). Reduction of Cardiovascular Risk by Regression of Electrocardiographic Markers of Left Ventricular Hypertrophy by the Angiotensin-Converting Enzyme Inhibitor Ramipril. *Circulation*.

[18] Muiesan ML, Salvetti M, Monteduro C, Bonzi B, Paini A, Viola S (2004). Left Ventricular Concentric Geometry during Treatment Adversely Affects Cardiovascular Prognosis in Hypertensive Patients. *Hypertension*.

[19] Verdecchia P (2003). Changes in cardiovascular risk by reduction of left ventricular mass in hypertension: a meta-analysis. *American Journal of Hypertension*.

[20] Muiesan ML, Salvetti M, Rizzoni D, Castellano M, Donato F, Agabiti-Rosei E (1995). Association of change in left ventricular mass with prognosis during long-term antihypertensive treatment. *Journal of Hypertension*.

[21] Kokkinos P, Myers J (2010). Exercise and Physical Activity. *Circulation*.

[22] Kokkinos P, Narayan P, Colleran J (1995). Effects of Exercise on Blood Pressure and Left Ventricular Hypertrophy in African-Americans with Severe Hypertension. *The New England Journal of Medicine*.

[23] Kokkinos P, Pittaras A, Narayan P, Faselis C, Singh S, Manolis A (2007). Exercise Capacity and Blood Pressure Associations with Left Ventricular Mass in Prehypertensive Individuals. *Hypertension*.

[24] Kokkinos P, Pittaras A, Manolis A, Panagiotakos D, Narayan P, Manjoros D (2006). Exercise capacity and 24-hr blood pressure in pre-hypertensive men and women. *American Journal of Hypertension*.

[25] Morganroth J, Maron B, Henry W, Epstein S (1975). Comparative Left Ventricular Dimensions in Trained Athletes. *Annals of Internal Medicine*.

[26] Pluim BM, Zwinderman AH, van der Laarse A, van der Wall EE (2000). The Athlete’s Heart. *Circulation*.

[27] Spirito P, Pelliccia A, Proschan MA, Granata M, Spataro A, Bellone P (1994). Morphology of the “athlete’s heart” assessed by echocardiography in 947 elite athletes representing 27 sports. *The American Journal of Cardiology*.

[28] Darling EA (1899). The Effects of Training. A Study of the Harvard University Crews. *The Boston Medical and Surgical Journal*.

[29] Henschen S.E (1899). Zusammenfassung. Skidlauf und skidwettlauf: eine medizinische sportstudie (66). *JENA, Fischer V.G., Upsala*.

[30] White PD (1918). The Pulse after a Marathon Race. *The Journal of the American Medical Association*.

[31] White PD (1942). Bradycardia in athletes, especially long-distance runners. *The Journal of the American Medical Association*.

[32] Arstila M, Koivikko A (1966). Electrocardiographic and vectorcardiographic signs of left and right ventricular hypertrophy in endurance athletes. *The Journal of Sports Medicine and Physical Fitness*.

[33] Chignon JC, Distel R, Courtois B, Leclerq J, Andrivet R (1969). Orientation of the analysis of electrical tracings regarding athletes. *The Journal of Sports Medicine and Physical Fitness*.

[34] Gott PH, Roselle HA, Crampton RS (1968). The athletic heart syndrome. Five-year cardiac evaluation of a champion athlete. *Archives of Internal Medicine*.

[35] Hanne-Paparo N, Wendkos MH, Brunner D (1971). T wave abnormalities in the electrocardiograms of top-ranking athletes without demonstrable organic heart disease. *American Heart Journal*.

[36] Hunt EA (1963). Electrocardiographic study of 20 champion swimmers before and after 110–yard sprint swimming competition. *Canadian Medical Association Journal*.

[37] Van Ganse W, Versee L, Eylenbosch W, Vuylsteek K (1970). The electrocardiogram of athletes Comparison with untrained subjects. *Heart*.

[38] Pelliccia A, Kinoshita N, Pisicchio C, Quattrini F, DiPaolo FM, Ciardo R (2010). Long-Term Clinical Consequences of Intense, Uninterrupted Endurance Training in Olympic Athletes. *Journal of the American College of Cardiology*.

[39] Pelliccia A, Maron BJ, Spataro A, Proschan MA, Spirito P (1991). The Upper Limit of Physiologic Cardiac Hypertrophy in Highly Trained Elite Athletes. *New England Journal of Medicine*.

[40] Sharma S, Maron BJ, Whyte G, Firoozi S, Elliott PM, McKenna WJ (2002). Physiologic limits of left ventricular hypertrophy in elite junior athletes. *Journal of the American College of Cardiology*.

[41] Fagard RH (1997). Ιmpact of different sports and training on cardiac structure and function. *Cardiology Clinics*.

[42] Nagashima J, Musha H, Takada H, Murayama M (2003). New upper limit of physiologic cardiac hypertrophy in Japanese participants in the 100-km ultramarathon. *Journal of the American College of Cardiology*.

[43] Reguero JJR, Cubero GI, de la Iglesia López J, Terrados N, Gonzalez V, Cortina R (1995). Prevalence and upper limit of cardiac hypertrophy in professional cyclists. *European Journal of Applied Physiology and Occupational Physiology*.

[44] Basavarajaiah S, Wilson M, Whyte G, Shah A, McKenna W, Sharma S (2008). Prevalence of Hypertrophic Cardiomyopathy in Highly Trained Athletes. *Journal of the American College of Cardiology*.

[45] Basavarajaiah S, Boraita A, Whyte G, Wilson M, Carby L, Shah A (2008). Ethnic Differences in Left Ventricular Remodeling in Highly-Trained Athletes. *Journal of the American College of Cardiology*.

[46] Rawlins J, Bhan A, Sharma S (2009). Left ventricular hypertrophy in athletes. *European Journal of Echocardiography*.

[47] Maron BJ (2003). Sudden Death in Young Athletes. *New England Journal of Medicine*.

[48] Basavarajaiah S, Wilson M, Junagde S, Jackson G, Whyte G, Sharma S (2006). Physiological left ventricular hypertrophy or hypertrophic cardiomyopathy in an elite adolescent athlete: role of detraining in resolving the clinical dilemma. *British Journal of Sports Medicine*.

[49] Pelliccia A, Maron BJ, De Luca R, Di Paolo FM, Spataro A, Culasso F (2002). Remodeling of Left Ventricular Hypertrophy in Elite Athletes after Long-Term Deconditioning. *Circulation*.

[50] Maron BJ, Pelliccia A (2006). The Heart of Trained Athletes. *Circulation*.

[51] Finocchiaro G, Sharma S (2016). Do endurance sports affect female hearts differently to male hearts. *Future Cardiology*.

[52] Schäfer D, Gjerdalen GF, Solberg EE, Khokhlova M, Badtieva V, Herzig D (2015). Sex differences in heart rate variability: a longitudinal study in international elite cross-country skiers. *European Journal of Applied Physiology*.

[53] Wheatley CM, Snyder EM, Johnson BD, Olson TP (2014). Sex differences in cardiovascular function during submaximal exercise in humans. *Springerplus*.

[54] Pelliccia A, Maron BJ, Culasso F, Spataro A, Caselli G (1996). Athlete’s heart in women. Echocardiographic characterization of highly trained elite female athletes. *The Journal of the American Medical Association*.

[55] Finocchiaro G, Dhutia H, D’Silva A, Malhotra A, Steriotis A, Millar L (2017). Effect of sex and sporting discipline on LV adaptation to exercise. *JACC: Cardiovasc Imaging*.

[56] Rawlins J, Carre F, Kervio G, Papadakis M, Chandra N, Edwards C (2010). Ethnic differences in physiological cardiac adaptation to intense physical exercise in highly trained female athletes. *Circulation*.

[57] Harper RW, Mottram PM (2009). Exercise-Induced Right Ventricular Dysplasia/Cardiomyopathy—an Emerging Condition Distinct from Arrhythmogenic Right Ventricular Dysplasia/Cardiomyopathy. *Heart, Lung and Circulation*.

[58] Abdulla J, Nielsen JR (2009). Is the risk of atrial fibrillation higher in athletes than in the general population? A systematic review and meta-analysis. *Europace*.

[59] Aizer A, Gaziano JM, Cook NR, Manson JE, Buring JE, Albert CM (2009). Relation of Vigorous Exercise to Risk of Atrial Fibrillation. *The American Journal of Cardiology*.

[60] Baldesberger S, Bauersfeld U, Candinas R, Seifert B, Zuber M, Ritter M (2008). Sinus node disease and arrhythmias in the long-term follow-up of former professional cyclists. *European Heart Journal*.

[61] Karjalainen J, Kujala UM, Kaprio J, Sarna S, Viitasalo M (1998). Lone atrial fibrillation in vigorously exercising middle aged men: case-control study. *British Medical Journal*.

[62] Molina L, Mont L, Marrugat J, Berruezo A, Brugada J, Bruguera J (2008). Long-term endurance sport practice increases the incidence of lone atrial fibrillation in men: a follow-up study. *Europace*.

[63] Mont L, Ruiz-Granell R, Martinez JG, Carmona JR, Fidalgo M, Cobo E (2008). Impact of anti-tachycardia pacing on atrial fibrillation burden when added on top of preventive pacing algorithms: results of the prevention or termination (POT) trial. *Europace*.

[64] Heidbüchel H, Anné W, Willems R, Adriaenssens B, Van de Werf F, Ector H (2006). Endurance sports is a risk factor for atrial fibrillation after ablation for atrial flutter. *International Journal of Cardiology*.

[65] Chodzko-Zajko WJ, Proctor DN, Fiatarone Singh MA, Minson CT, Nigg CR, Salem GJ (2009). Exercise and Physical Activity for Older Adults. *Medicine & Science in Sports & Exercise*.

[66] Mont L, Elosua R, Brugada J (2009). Endurance sport practice as a risk factor for atrial fibrillation and atrial flutter. *Europace*.

[67] Heidbuchel H, Prior DL, La Gerche A (2012). Ventricular arrhythmias associated with long-term endurance sports: what is the evidence. *British Journal of Sports Medicine*.

[68] La Gerche A, Burns AT, Mooney DJ, Inder WJ, Taylor AJ, Bogaert J (2012). Exercise-induced right ventricular dysfunction and structural remodelling in endurance athletes. *European Heart Journal*.

[69] O’Keefe JH, Patil HR, Lavie CJ, Magalski A, Vogel RA, McCullough PA (2012). Potential Adverse Cardiovascular Effects from Excessive Endurance Exercise. *Mayo Clinic Proceedings*.

[70] Biffi A, Maron BJ, Di Giacinto B, Porcacchia P, Verdile L, Fernando F (2008). Relation between Training-Induced Left Ventricular Hypertrophy and Risk for Ventricular Tachyarrhythmias in Elite Athletes. *The American Journal of Cardiology*.

[71] Faselis C, Kokkinos P, Tsimploulis A, Pittaras A, Myers J, Lavie CJ (2016). Exercise Capacity and Atrial Fibrillation Risk in Veterans. *Mayo Clinic Proceedings*.

[72] Pittaras AE, Faselis C, Samuel IBH, Doumas M, Grassos H, Murphy R (2022). Exercise capacity and atrial fibrillation risk in 459,592 hypertensive US veterans: a cohort study. *Journal of the American College of Cardiology*.

[73] Chobanian AV, Bakris GL, Black HR, Cushman WC, Green LA, Izzo JL (2003). The Seventh Report of the Joint National Committee on Prevention, Detection, Evaluation, and Treatment of High Blood Pressure: the JNC 7 report. *The Journal of the American Medical Association*.

[74] Collins R, Peto R, Godwin J, Macmahon S (1990). Blood pressure and coronary heart disease. *The Lancet*.

[75] Cornelissen VA, Fagard RH (2005). Effects of Endurance Training on Blood Pressure, Blood Pressure–Regulating Mechanisms, and Cardiovascular Risk Factors. *Hypertension*.

[76] Pescatello LS, Franklin BA, Fagard R, Farquhar WB, Kelley GA, Ray CA (2004). Exercise and Hypertension. *Medicine & Science in Sports & Exercise*.

[77] Huai P, Xun H, Reilly KH, Wang Y, Ma W, Xi B (2013). Physical Activity and Risk of Hypertension. *Hypertension*.

[78] Whelton SP, Chin A, Xin X, He J (2002). Effect of Aerobic Exercise on Blood Pressure. *Annals of Internal Medicine*.

[79] Blair SN, Kohl HW, Barlow CE, Gibbons LW (1991). Physical fitness and all-cause mortality in hypertensive men. *Annals of Medicine*.

[80] Faselis C, Doumas M, Panagiotakos D, Kheirbek R, Korshak L, Manolis A (2012). Body mass index, exercise capacity, and mortality risk in male veterans with hypertension. *American Journal of Hypertension*.

[81] Kokkinos P, Manolis A, Pittaras A, Doumas M, Giannelou A, Panagiotakos DB (2009). Exercise Capacity and Mortality in Hypertensive Men with and without Additional Risk Factors. *Hypertension*.

[82] Kokkinos P, Myers J, Doumas M, Faselis C, Manolis A, Pittaras A (2009). Exercise Capacity and all-Cause Mortality in Prehypertensive Men. *American Journal of Hypertension*.

[83] Myers J, Prakash M, Froelicher V, Do D, Partington S, Atwood JE (2002). Exercise Capacity and Mortality among Men Referred for Exercise Testing. *New England Journal of Medicine*.

[84] Collins R, Peto R, MacMahon S, Godwin J, Qizilbash N, Collins R (1990). Blood pressure, stroke, and coronary heart disease. *The Lancet*.

[85] MacMahon S, Peto R, Cutler J, Collins R, Sorlie P, Neaton J (1990). Blood pressure, stroke, and coronary heart disease. Part 1, Prolonged differences in blood pressure: prospective observational studies corrected for the regression dilution bias. *Lancet*.

[86] Baglivo HP, Fabregues G, Burrieza H, Esper RC, Talarico M, Esper RJ (1990). Effect of moderate physical training on left ventricular mass in mild hypertensive persons. *Hypertension*.

[87] Hinderliter A, Sherwood A, Gullette ECD, Babyak M, Waugh R, Georgiades A (2002). Reduction of Left Ventricular Hypertrophy after Exercise and Weight Loss in Overweight Patients with Mild Hypertension. *Archives of Internal Medicine*.

[88] Turner MJ, Spina RJ, Kohrt WM, Ehsani AA (2000). Effect of Endurance Exercise Training on Left Ventricular Size and Remodeling in Older Adults with Hypertension. *The Journals of Gerontology Series a: Biological Sciences and Medical Sciences*.

[89] Rinder MR, Spina RJ, Peterson LR, Koenig CJ, Florence CR, Ehsani AA (2004). Comparison of effects of exercise and diuretic on left ventricular geometry, mass, and insulin resistance in older hypertensive adults. *American Journal of Physiology-Regulatory, Integrative and Comparative Physiology*.

[90] Palatini P, Visentin P, Dorigatti F, Guarnieri C, Santonastaso M, Cozzio S (2009). Regular physical activity prevents development of left ventricular hypertrophy in hypertension. *European Heart Journal*.

[91] Reid CM, Dart AM, Dewar EM, Jennings GL (1994). Interactions between the effects of exercise and weight loss on risk factors, cardiovascular haemodynamics and left ventricular structure in overweight subjects. *Journal of Hypertension*.

[92] Klingbeil AU, Schneider M, Martus P, Messerli FH, Schmieder RE (2003). A meta-analysis of the effects of treatment on left ventricular mass in essential hypertension. *The American Journal of Medicine*.

[93] Dahlöf B, Devereux RB, Kjeldsen SE, Julius S, Beevers G, de Faire U (2002). Cardiovascular morbidity and mortality in the Losartan Intervention for Endpoint reduction in hypertension study (LIFE): a randomised trial against atenolol. *The Lancet*.

[94] Kokkinos P, Chrysohoou C, Panagiotakos D, Narayan P, Greenberg M, Singh S (2006). Beta-Blockade Mitigates Exercise Blood Pressure in Hypertensive Male Patients. *Journal of the American College of Cardiology*.

[95] Kokkinos PF, Narayan P, Fletcher RD, Tsagadopoulos D, Papademetriou V (1997). Effects of aerobic training on exaggerated blood pressure response to exercise in African-Americans with severe systemic hypertension treated with indapamide ± verapamil ± enalapril. *The American Journal of Cardiology*.

[96] Nelson ME
, Rejeski WJ, Blair SN, Duncan PW, Judje JO, King AC (2007). Physical Activity and Public Health in Older Adults. *Medicine & Science in Sports & Exercise*.

[97] Visseren F, Mach F, Smulders Y, Carballo D, Koskinas K, Bäck M (2021). 2021 ESC Guidelines on cardiovascular disease prevention in clinical practice: Developed by the Task Force for cardiovascular disease prevention in clinical practice with representatives of the European Society of Cardiology and 12 medical societies. With the special contribution of the European Association of Preventive Cardiology (EAPC). *European Heart Journal*.

[98] Piercy KL, Troiano RP, Ballard RM, Carlson SA, Fulton JE, Galuska DA (2018). The Physical Activity Guidelines for Americans. *The Journal of the American Medical Association*.

